# Durable response to anti-PD-1 immunotherapy in epithelioid angiomyolipoma: a report on the successful treatment of a rare malignancy

**DOI:** 10.1186/s40425-018-0415-x

**Published:** 2018-10-01

**Authors:** Michael Lattanzi, Fang-Ming Deng, Luis A. Chiriboga, Alisa N. Femia, Shane A. Meehan, Gopa Iyer, Martin H. Voss, Yuliya Sundatova, William C. Huang, Arjun V. Balar

**Affiliations:** 1 Department of Medicine, NYU Langone Health, New York, NY USA; 2 Department of Pathology, NYU Langone Health, New York, NY USA; 3 Department of Urology, NYU Langone Health, New York, NY USA; 4 Ronald O. Perelman Department of Dermatology, NYU Langone Health, New York, NY USA; 5 Laura and Isaac Perlmutter Cancer Center, NYU Langone Health, New York, NY USA; 6 Department of Medicine, Memorial Sloan-Kettering Cancer Center, New York, NY USA; 7 Genitourinary Medical Oncology Program, NYU School of Medicine, Laura and Isaac Perlmutter Cancer Center, NYU Langone Health, 160 East 34th Street, 10th Floor, New York, NY 10016 USA

**Keywords:** Immunotherapy, Angiomyolipoma, PEComa, Nivolumab, PD-1, PD-L1, Tuberous sclerosis, TSC2, mTOR, Everolimus

## Abstract

**Background:**

Malignant angiomyolipoma is an uncommon tumor of the class of perivasciular epithelioid cell neoplasms (PEComas). These tumors are characteristically driven by deleterious mutations in the tumor suppressors *TSC1* and *TSC2*, whose gene products typically act to inhibit mTOR. There are several cases of malignant angiomyolipoma which exhibit transient responses to mTOR inhibitors, forming the basis of current practice guidelines in malignant PEComa. However the tumors ultimately acquire resistance, and there is no well-established second-line option. Despite the increasing prevalence of immunotherapy across a wide range of solid tumors, little is known about the immune infiltrate and PD-L1 expression of angiomyolipoma. Furthermore, there is no reported case on the treatment of malignant angiomyolipoma with an immune checkpoint inhibitor.

**Case presentation:**

A 38 year-old man presented with gross hematuria and was diagnosed with renal epithelioid angiomyolipoma. Despite surgical resection, the tumor recurred and metastasized. Targeted genomic sequencing revealed a deleterious mutation in *TSC2*, and the patient was treated with the mTOR inihbitor everolimus. The patient went on to have a partial response but ultimately progressed. He was then treated with the anti-PD-1 immune checkpoint inhibitor nivolumab, and achieved a durable near-complete response which is ongoing after two years of treatment. Immunohistochemical staining of tumor tissue revealed strong PD-L1 expression and a brisk T-cell infiltrate.

**Conclusions:**

We report on the first durable systemic treatment of malignant epithelioid angiomyolipoima with the use of PD-1 antibody nivolumab. Given the absence of prospective clinical trials in this exceedingly rare disease, particularly in the second-line setting, immune checkpoint inhibitors like nivolumab should be considered.

## Background

Angiomyolipomas (AMLs) are neoplasms thought to arise from pericites, hence belonging to the family of perivasciular epithelioid cell neoplasms (PEComas). Though associated with tuberous sclerosis complex (TSC), most AMLs arise de novo, in the absence of germline *TSC1* or *TSC2* mutations [1]. There exist two well-described histologic variants: classical and epithelioid. Although the vast majority of AMLs are benign, a minority of epithelioid AML (EAML) may become malignant and have been reported to metastasize [2]. The exact prevalence of malignant EAML is not well characterized; however, it likely falls below 1:300,000 [3, 4]. Given its rarity, there is no established treatment for unresectable or metastatic EAML.

TSC is a genetic syndrome characterized by multisystem tumor development, including renal angiomyolipoma. Most patients harbor pathogenic germline loss-of-function mutations in *TSC1* or *TSC2* [5], whose wild-type gene products inhibit mammalian target of rapamycin (mTOR) complex 1 (mTORC1) [6, 7]. This observation prompted a phase III placebo-controlled trial demonstrating significant tumor regression of TSC-associated AML with everolimus [8], a United States Food and Drug Administration (FDA)-approved allosteric inhibitor of mTORC1. Somatic mutations in *TSC1* and *TSC2* also contribute to tumor growth via unopposed mTOR signaling, and sporadic AML is similarly characterized by somatic loss-of-function alterations in *TSC2* [9]. Multiple reports detail responses to mTOR inhibitors among tumors harboring *TSC1* or *TSC2* mutations [7, 10], including PEComa, not otherwise specified [11], and sporadic AML [12], though DNA sequencing was not reported. Based on these responses, clinical practice guidelines for malignant PEComa currently emphasize the use of mTOR inhibitors such as everolimus [13]. However, despite an initial response to rapalog therapy, virtually all patients ultimately develop progressive disease, and there is no well-established second-line treatment.

Nivolumab is a fully humanized monoclonal IgG4 antibody that targets the programmed death 1 (PD-1) receptor, an immune checkpoint expressed on exhausted effector T lymphocytes, and prevents binding by its activating ligand PD-L1, leading to reinvigoration of anti-tumor immunity [14]. Nivolumab is FDA-approved for melanoma, renal cell carcinoma, and urothelial bladder cancer, among other solid tumors. Although tumor PD-L1 expression is associated with response [15], no biomarker of response has been rigorously validated. Additionally, immune checkpoint inhibitors are associated with the development and/or exacerbation of autoimmunity [15], and such immune-related toxicities may correlate with enhanced clinical efficacy [16].

Given the lack of data concerning the treatment of this rare cancer, we report a case of metastatic EAML harboring a deleterious *TSC2* mutation. The patient exhibited a transient response to everolimus, but ultimately progressed. He subsequently achieved a significant and durable response to nivolumab. To the best of our knowledge, this is the first report on the treatment of malignant EAML with immunotherapy.

## Case presentation

**Fig. 1:**
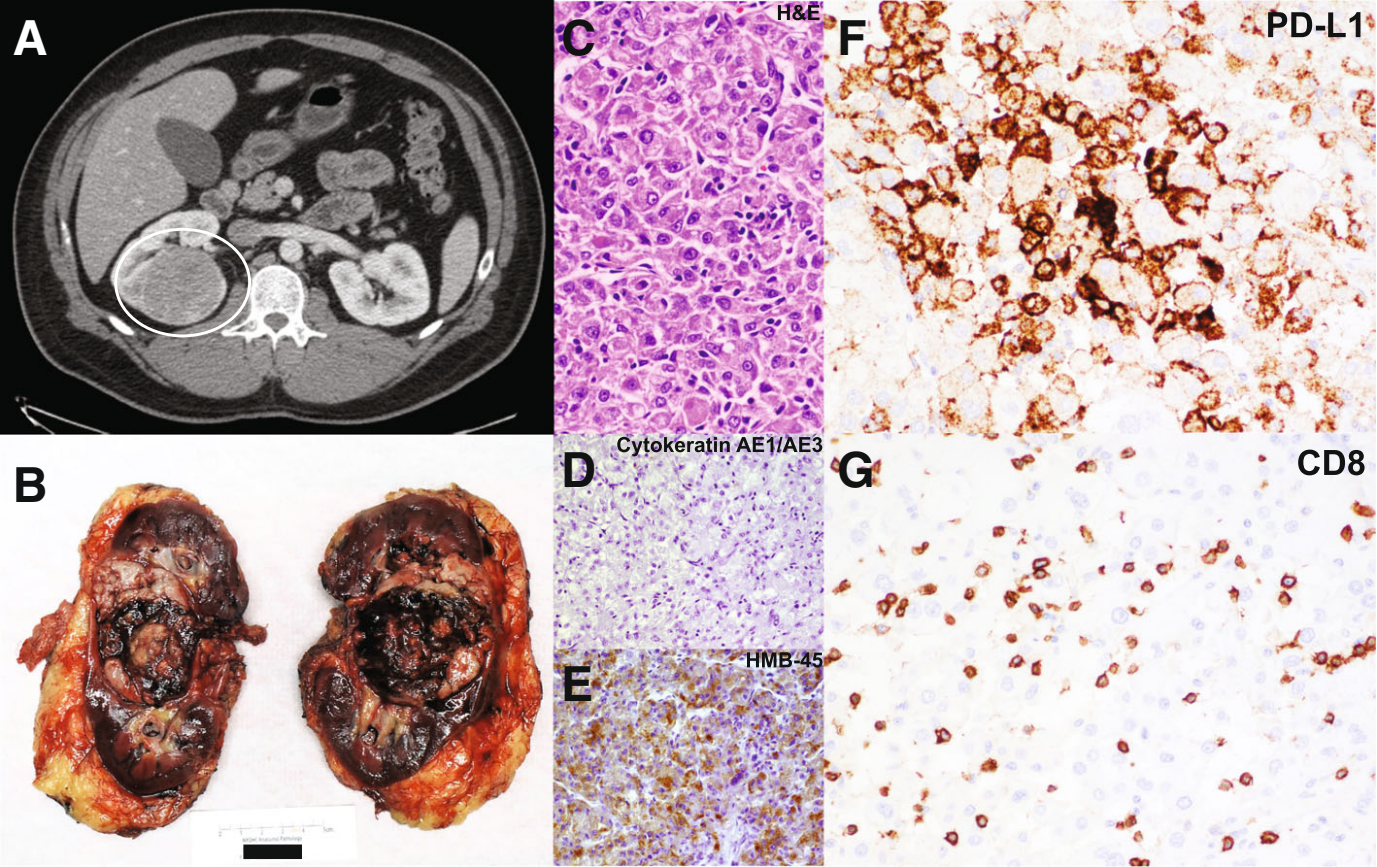
**a** CT Urogram demonstrating the primary right renal mass, (**b**) Gross pathology demonstrating the resected perihilar tumor with central necrosis, (**c**) H&E stain demonstrating angiomyolipoma with a substantial epithelial component, (**d**) Immunohistochemical stain negative for cytokeratin AE1/AE3, (**e**) Immunohistochemical stain positive for HMB-45, (**f**) Immunohistochemical stain positive for PD-L1 (> 50% of cells), (**f**) Immunohistochemical stain positive for T lymphocyte marker CD8

A 38 year-old man with vitiligo and hypothyroidism initially presented in 2011 with gross hematuria. Diagnostic imaging (Fig. 1a) revealed a 6-cm renal mass concerning for malignancy, for which he underwent a right radical nephrectomy at the recommendation of his treating urologic oncologist (WCH). Gross pathology (Fig. 1b) revealed a 6 × 5-cm encapsulated hilar mass with hemorrhage and central necrosis. The mass was limited to the renal parenchyma, without evidence of renal sinus or vascular invasion, and surgical margins were negative for tumor cells. Histologic sections (Fig. 1c) demonstrated sheets of epithelioid cells with sarcomatoid and rhabdoid features as well as round, polygonal cells with pleomorphic nuclei and prominent nucleoli. Mitotic figures were visualized at a rate of approximately three per high-powered field. Immunohistochemical staining (Fig. 1d-e) revealed tumor cell positivity for: HMB45, melan-A, carbonic anhydrase IX, and to a lesser extent, Cam5.2, vimentin and SMA (cytoplasmic), and negativity for: EMA, keratins (AE1/3), CK7, CK20, P63, Pax-2, AMACAR, S-100, and CD10. Based on these histo-pathologic features, the patient was diagnosed with primary EAML.

The patient had an uneventful course for the next 3 years until April, 2014, when surveillance imaging detected an asymptomatic 13-cm renal fossa mass for which he underwent repeat surgical resection. Surgical pathology confirmed recurrent EAML, again with negative margins. The patient’s tumor recurred again in October, 2014, prompting a third surgical resection. Pathologic evaluation this time demonstrated indeterminate margins, prompted referral to medical oncology for further management.

December, 2014 surveillance imaging obtained by the treating medical oncologist (AVB) demonstrated new retroperitoneal and pelvic implants consistent with metastatic EAML. The patient’s tumor DNA was subjected to FoundationOne® targeted next-generation sequencing [17], which revealed four oncogenic alterations: truncating mutations in TP53 and APC, a frameshift mutation in *ATRX*, and a deletion in *TSC2*, specifically, TSC2 H1746_R1751del, which has been reported both as a somatic variant in AML [18] and as a germline mutation in TSC [19]. Of note, the FoundationOne® assay demonstrated no genomic alterations in the four genes encoding key DNA mismatch repair proteins: *MSH2*, *MSH6*, *PMS2*, or *MLH1*.

**Fig. 2:**
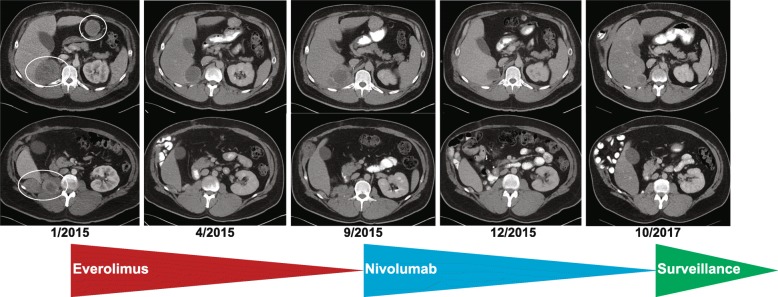
Schematic representation of radiographic tumor burden over time indicating (left-to-right): baseline pre-everolimus scan, best response to everolimus, progression on everolimus and baseline pre-nivolumab scan, initial post-nivolumab response, and continued response at the time of nivolumab discontinuation

Based on the *TSC2* deletion, the patient was initiated on everolimus in January, 2015. Imaging at 3 months (Fig. 2) demonstrated marked decrease in size of the majority of the soft tissue masses throughout the right nephrectomy bed, retroperitoneum, and mesentery, and no new sites of disease. The patient remained clinically asymptomatic for 8 months, until he noted unintended weight loss in September, 2015. Imaging demonstrated slight enlargement of the dominant right renal fossa mass (Fig. 2), which in the context of progressive anemia, was interpreted as disease progression. Everolimus was discontinued, and the patient was referred for a treatment-directed biopsy for consideration of a clinical trial.

He underwent a biopsy of the dominant 6 cm retroperitoneal mass, from which DNA was isolated and subjected to paired tumor-germline next-generation sequencing via MSK-IMPACT [20], which confirmed the absence of a *TSC2* germline mutation. However, no new somatic variants were identified to explain the tumors’ acquired resistance, and he was not eligible for any clinical trials. Given the well-known activity of anti-PD-1 checkpoint inhibition across a range of advanced solid tumors, including renal cell carcinoma [21], the patient was offered a trial of off-label nivolumab via the Bristol-Myers Squibb Expanded Access program, and he began treatment in October, 2015. After two cycles of nivolumab (administered at 3 mg/kg IV every 2 weeks), the patient felt well, and resolution of his anemia suggested possible clinical benefit. Imaging after 5 cycles demonstrated responding disease (Fig. 2).

**Fig. 3:**
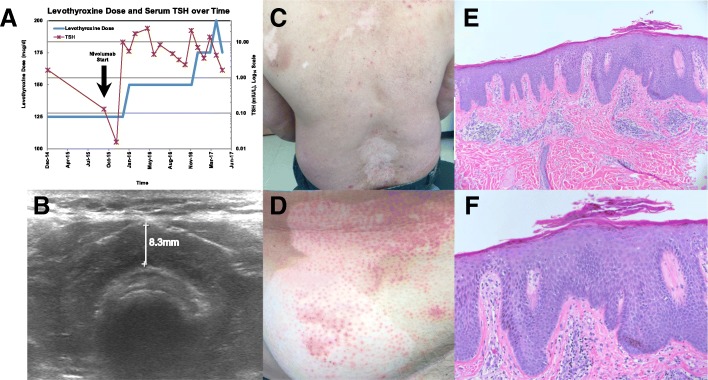
**a** Graph of the patient’s prescribed daily dose of levothyroxine and serum thyroid stimulating hormone (TSH) over time relative to initiation of nivolumab, (**b**) Archival thyroid ultrasound demonstrating enlarged, heterogeneous thyroid gland suggestive of chronic thyroiditis, (**c**) Distribution of psoriasis involving the patient’s back, (**d**) Representative scaly, well-demarcated erythematous papules overlying the right lower abdomen predominantly within an area of vitiligo, (**e**) Low-resolution and (**f**) High-resolution histologic sections of cutaneous biopsy with H&E staining demonstrating a thin granular layer, confluent parakeratosis with collections of neutrophils, and dilated capillaries throughout the papillary dermis, consistent with psoriasis

Nivolumab was well-tolerated, with the exception of immune-related exacerbation of pre-existing hypothyroidism (Fig. 3a-b) after 2 months of therapy, and immune-related pruritic cutaneous eruption predominantly within areas of pre-existing vitiligo (Fig. 3c-d), occurring after 18 months of treatment. Over the course of nivolumab therapy, the patient required increasing doses of levothyroxine to maintain a euthyroid state. An archival thyroid ultrasound reveals an enlarged heterogenous thyroid gland suggestive of possible autoimmune thyroiditis. With regard to cutaneous toxicity, the patient was referred to dermatology (ANF), and a skin biopsy was obtained of an involved area on the upper back. Histologic evaluation by the dermatopathologist (SAM) revealed a thin granular layer, confluent parakeratosis with collections of neutrophils, and dilated capillaries throughout the papillary dermis, consistent with psoriasis (Fig. 3e-f).

**Fig. 4:**
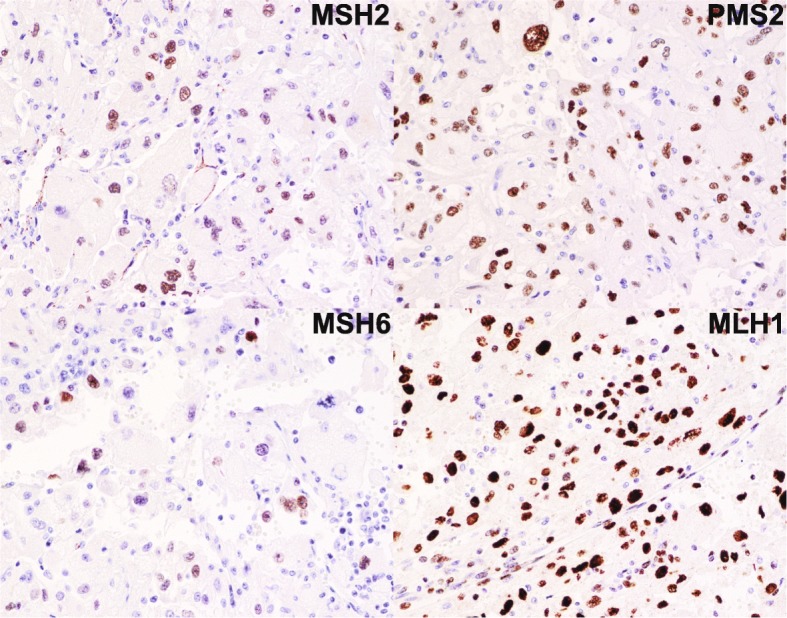
Immunohistochemical stains for key DNA mismatch repair proteins revealing preserved expression of MSH2, MSH6, PMS2, and MLH1, thereby confirming DNA mismatch repair proficiency

Although these toxicities were not dose-limiting, the patient had already completed 2 years of therapy [22], and nivolumab was therefore discontinued. The most recent imaging at the time of discontinuation in November, 2017 demonstrates continued response and interval calcification of his intra-abdominal tumors (Fig. 2). Archival tissue derived from the initial surgical resection was analyzed for PD-L1 and CD8 expression via immunohistochemistry (Fig. 1f-g) utilizing a modified Agilent/Dako 22C3 pharmaDX kit, revealing high PD-L1 expression (> 50% of cells) and a brisk CD8+ T cell infiltrate. Finally, immunohistochemical analysis demonstrated preserved expression of key DNA mismatch repair (MMR) proteins MSH2, MSH6, PMS2, and MLH1 [23], thus confirming MMR proficiency (Fig. 4).

## Discussion

Current approaches to treatment for the sporadic form of recurrent or metastatic EAML are derived from anecdotal reports of efficacy with mTOR inhibitors [11, 24–27]. Furthermore, although Wagner, et al. [11] reported *TSC1* loss in PEComa, genomic sequencing of *TSC1* and *TSC2* was not performed in these cases, as they predate the widespread use of next-generation sequencing, and the histologic subtype (e.g. angiomyolipoma) was not specified. Nonetheless, despite an initial response to rapalog therapy, virtually all tumors ultimately developed resistance. In addition to mTOR inhibitors, tumor responses have been observed with the use of chemotherapy. Cibas, et al. reported a partial response to doxorubicin [28], and others have reported various combinations of cisplatin, cyclophosphamide, dacarbazine, and ifosfamide [29], with modest results and no reported long-term responses.

Cumulatively, *TSC1* and *TSC2* alterations occur in approximately 5% of all solid tumors, including 4.7% of the MSK-IMPACT cohort [20] and 5.2% of the corresponding TCGA cohort [30]. In the COSMIC database [31], TSC2 H1746_R1751del has been previously identified in six tumors, including two renal angiomyolipomas, as reported by Qin, et al. [18]. However, neither of these cases was predominantly epithelioid; neither was reportedly malignant; and one carried a diagnosis of TSC. Of note, genomic sequencing reported by Wagle, et al. identified a gain-of-function mutation in *MTOR* as the etiology of everolimus resistance in *TSC2*-mutant thyroid carcinoma. However, no post-treatment alterations were identified to explain the acquired resistance in the present case of EAML.

In contrast to the transient responses to everolimus both in our case and others’, we report the first durable response to any agent in this rare malignancy with the use of the PD-1 antibody nivolumab. To the best of our knowledge, this case is the only report on the use of immunotherapy in the management of angiomyolipoma of any primary site or histologic variant. Immunohistochemistry revealed high PD-L1 expression (> 50% of cells), and brisk cytotoxic T cell infiltrate (Fig. 1f-g). The striking PD-L1 expression in a tumor responsive to anti-PD-1 checkpoint inhibitor immunotherapy is consistent with previous reports supporting a correlation between PD-L1 expression and response to anti-PD-1 and/or anti-PD-L1 immunotherapy. Specifically, in urothelial carcinoma [32] and non-small cell lung adenocarcinoma [33], there is a strong relationship between PD-L1 expression and response to nivolumab. PD-L1 positivity in this case of nivolumab-responsive EAML may represent a predictive biomarker in this histology as in other cancers, however, this case is the only report of PD-L1 expression in EAML and further study is needed. In melanoma, lymphocytic infiltrate is associated with increased likelihood of response to anti-PD-1 checkpoint inhibitor [34]. The presence of a brisk CD8+ T cell infiltrate suggests intrinsic anti-tumor immunologic activity and may also represent a biomarker of response in EAML. Interestingly, although this patient initiated nivolumab prior to the FDA’s histology-independent approval for PD-1 antibodies in DNA mismatch repair deficient (dMMR) tumors, subsequent immunohistochemical analysis demonstrated MMR proficiency, and this patient would not have been a candidate for nivolumab on the basis of MMR status.

Also of interest is our patient’s history of pre-existing as well as treatment-related autoimmunity. The development of immune-related adverse events has been correlated to clinical benefit with immunotherapy among melanoma patients, a subset of whom develop vitiligo [16]. In the present patient, the development of anti-PD-1-related psoriaform dermatitis and an exacerbation of presumed autoimmune thyroiditis are highly suggestive of a systemic immune activation secondary to anti-PD-1 immunotherapy.

## Conclusions

In this case of malignant EAML – a rare disease with no standard of care – we report a durable near-complete response to the anti-PD-1 immune checkpoint inhibitor, nivolumab. This case highlights the cross-histologic efficacy of immune checkpoint inhibition, particularly in tumors with high PD-L1 expression and brisk lymphocytic infiltrate. In the absence of prospective clinical trials, nivolumab should be considered for use in other patients with recurrent or metastatic EAML who have exhausted traditional therapeutic options.

## Abbreviations

AML: Angiomyolipoma
EAML: Epithelioid angiomyolipoma
FDA: Food and Drug Administration
MMR: Mismatch repair
MTOR: Mammalian target of rapamycin
PD-1: Programmed death receptor 1
PD-L1: Programmed death receptor ligand 1
TSC: Tuberous sclerosis complex
